# Deformability Assessment of Waterborne Protozoa Using a Microfluidic-Enabled Force Microscopy Probe

**DOI:** 10.1371/journal.pone.0150438

**Published:** 2016-03-03

**Authors:** John S. McGrath, Jos Quist, James R. T. Seddon, Stanley C. S. Lai, Serge G. Lemay, Helen L. Bridle

**Affiliations:** 1 Institute of Biological Chemistry, Biophysics and Bioengineering, School of Engineering and Physical Sciences, Heriot-Watt University, Edinburgh, EH14 4AS, United Kingdom; 2 Nanoionics group, MESA+ Institute for Nanotechnology, University of Twente, PO BOX 217, 7500 AE Enschede, The Netherlands; LAAS-CNRS, FRANCE

## Abstract

Many modern filtration technologies are incapable of the complete removal of *Cryptosporidium* oocysts from drinking-water. Consequently, *Cryptosporidium*-contaminated drinking-water supplies can severely implicate both water utilities and consumers. Existing methods for the detection of *Cryptosporidium* in drinking-water do not discern between non-pathogenic and pathogenic species, nor between viable and non-viable oocysts. Using FluidFM, a novel force spectroscopy method employing microchannelled cantilevers for single-cell level manipulation, we assessed the size and deformability properties of two species of *Cryptosporidium* that pose varying levels of risk to human health. A comparison of such characteristics demonstrated the ability of FluidFM to discern between *Cryptosporidium muris* and *Cryptosporidium parvum* with 86% efficiency, whilst using a measurement throughput which exceeded 50 discrete oocysts per hour. In addition, we measured the deformability properties for untreated and temperature-inactivated oocysts of the highly infective, human pathogenic *C*. *parvum* to assess whether deformability may be a marker of viability. Our results indicate that untreated and temperature-inactivated *C*. *parvum* oocysts had overlapping but significantly different deformability distributions.

## Introduction

*Cryptosporidium* is a problematic agent of waterborne disease, which is highly infectious [1] and able to persist for several months in treated water supplies [2]. This protozoan pathogen is capable of eluding conventional disinfection procedures and also many modern, 99.99% efficient filtration technologies [3,4]. With the global demand for potable drinking-water continuing to increase, the incidence of cryptosporidiosis is predicted to rise by the World Health Organization [5].

As the ingestion of less than ten oocysts can cause a significant infection in healthy individuals [6], and because *Cryptosporidium*-contaminated supplies often contain mixed species [7], it is important that a revised or improved detection method is capable of discrimination at the single-oocyst level. The existing method, EPA 1623.1, recovers and detects parasites within the genus *Cryptosporidium* by filtration, immunomagnetic separation and immunofluorescence assay microscopy, but gives no indication of viability or species [8]. A rapid, efficient method of discerning between non-infectious oocysts and species which are capable of causing human infection would aid in better assessing the risk posed to human health by contaminated samples. For example, regulatory actions would no longer be necessary if contamination poses no significant risk, such as in a situation where a contamination event was due to the presence of a non-human pathogenic species. This would save numerous resources for water utilities.

In this paper, we investigate whether the biomechanical properties and sizes of oocysts vary depending on species and viability. We also assess whether temperature-based inactivation causes a change in deformability, which in turn provides a marker for (lack of) infectivity. The *Cryptosporidium* oocyst wall is considered deformable [9,10], which contributes to its ability to elude filtration. Should oocyst size/deformability vary across the genus due to differences in structure and composition, methods could be designed to exploit these properties for species-level discrimination/sorting of oocysts, *e*.*g*., deformability-based [11,12] and/or size-based [13,14] separation microfluidics may be a useful tool. Additionally, if oocyst deformability varies between viable and non-viable sub-populations of the same species due to the degradation of wall and internal structures after loss of viability, such methods could also potentially be used for viability sorting of oocysts. Therefore, it is of considerable interest to quantify the variation in deformability between *Cryptosporidium* species and between viable and non-viable sub-populations of human-pathogenic species in order to optimise treatment and detection approaches.

Previous studies [9,15–18] have used colloidal probe microscopy to study the outer wall of *Cryptosporidium parvum*. In these studies, silica particles were used as a colloidal probe to study oocysts immobilised on a surface. These studies focussed mainly on the colloidal properties of the oocysts (*e*.*g*., interactions with sand particles), but provided little information into the overall deformability of oocysts. Considine *et al*. [15] reported highly variable effective spring constants for the deformation of a small sample (*n* = 5) of *C*. *parvum* oocysts, but no comparison was made between different *Cryptosporidium* species and between viable and non-viable oocysts. A force microscopy tool that can routinely measure the overall deformability of oocysts of different species and/or viability status is therefore desirable.

Here, we use a FluidFM [19,20], a force microscopy platform for single cell analysis, which employs a microchannelled cantilever with an aperture to immobilise, manoeuvre and release single cells. FluidFM has been used for several purposes including the quantification of bacterial adhesion [21] and monitoring the deformation of beating cardiac cells [22]. In this work, a FluidFM was used for force spectroscopy on relatively large numbers of individual oocysts in order to assess the effect of temperature-based inactivation treatments on *C*. *parvum* and the interspecies deformability of *C*. *parvum vs*. *C*. *muris*. Immobilisation or release was achieved by applying suction or positive pressure, and single oocysts were measured in rapid sequence and in different orientations, demonstrating a significant advance over conventional colloidal probe microscopy.

## Materials and Methods

### Oocyst treatment

*C*. *parvum* and *C*. *muris* oocysts were obtained suspended in phosphate-buffered saline (PBS) from Waterborne Inc. (New Orleans, LA) in stock samples containing ~1250 oocysts μL^-1^ and were stored at 4°C. In order to assess inactivated oocysts, heat treatment was performed by incubating the prepared sample for 10 min at 70°C in a heating block (HLC Cooling-ThermoMixer MKR 23, Ditabis, Germany). For the freeze-thawing treatment, the oocysts were subjected to one cycle of freezing at -18°C for 18 hr.

### Force spectroscopy

A FluidFM (Cytosurge AG, Glattbrug, Switzerland) was used for force spectroscopy. In this instrument, a FlexAFM 5 scan head (Nanosurf AG, Liestal, Switzerland) is mounted on a Zeiss Axio Observer.Z1 inverted microscope. The instrument was placed on a passive vibration isolation table. FluidFM Micro Pipettes (Cytosurge)–microchannelled force microscopy cantilevers which are actuated using a pressure controller–were used to pick up and measure the deformability of individual oocysts. Cantilevers had either 2 μm (for *C*. *parvum*) or 4 μm (for *C*. *muris*) apertures, considering the different sizes of the sampled species. For each measurement set, an aliquot of 4 μL of suspension (~5000 oocysts) was pipetted into a glass petri dish containing 7–8 mL of PBS, ensuring that the cantilever tip was fully immersed in liquid.

CyUI software (Cytosurge) was used for simultaneous microscopy, pressure control and force spectroscopy. Force spectroscopy measurements were performed by approaching the substrate towards the cantilever at a speed of 600 nm/s, whilst keeping the oocyst attached to the cantilever by suction. Before a series of experiments, the spring constant of the unfilled cantilever (typical value ~2 N/m) was measured in air by the Sader method [23] and corrected for the 11° angle of the cantilever [24], and the deflection sensitivity of the water-filled cantilever was measured in buffer solution through hard contact with the glass substrate. Measurements were conducted at room temperature (21°C).

### Data analysis

Matlab (R2013a) was used to convert deflection measurements to force-distance curves and to fit models. To reduce noise artefacts, the curves were binned over 40 data points for *C*. *parvum* and 16 data points for *C*. *muris* (original sample frequencies 6000 and 2400 Hz, respectively), resulting in one data point per 4 nm. Piezo displacement was subtracted from the initial cantilever-substrate separation and corrected for cantilever deflection to obtain the height of the (compressed) oocyst at each data point. The point of first contact was estimated by using a 0.1 nN threshold on the approach curves, the corresponding cantilever-substrate separation provided an indication of oocyst height (*i*.*e*., the difference in distance at the point of first contact with and without an oocyst attached). If the force-distance curve contained a kink, as described further below, the data points on and above the kinks were not considered in further analysis. To obtain deformability (effective spring constant) values, a linear fit was applied to the upper part of the force-distance curves. At least 8 adjacent data points (32 nm) were used for the linear fit. See S1 Fig for a schematic overview of the data analysis procedure.

### Statistical analysis

Data sets of height and deformability of untreated, heat-treated and freeze-thawed *C*. *parvum* were tested for normal distribution and equal variance using SPSS Statistics software (Version 22, IBM) to assess the suitability of statistical tests intended for parametric data. Specifically, data sets were tested for normality using both the Kolmogorov-Smirnov test and Shapiro-Wilk test–due to the common presence of skewness in distribution of spring constant values, data are commonly discussed using medians and quartiles, unless otherwise indicated. Data was tested for homogeneity of variance using Levene’s F test. Consequently, the Welch’s F test and Games-Howell test (typically used in conjunction with Welch’s F test for *post-hoc* analysis) were used to compare the deformability distributions of the oocyst populations. A one-way ANOVA was used for comparison of height data, since the homogeneity of variances assumption was met for this data. Significance was recorded at the 0.05 confidence level (*p* < 0.05).

A script was written using Matlab (R2013a) to quantify the discrimination efficiency of the sampled populations with regards to height and/or spring constant distributions where applicable (*i*.*e*., it was not necessary to quantify overlap of the height distributions for untreated *vs*. temperature-inactivated *C*. *parvum* because all populations showed similarity in values). Non-parametric methods were used for estimation due to the non-normal distribution of some data sets. A kernel probability density function (PDF) was fitted to the height or spring constant data for two different populations in order to define the overlapping area of the fitted distributions (see S2 Fig for overview of procedure). The probability that a subject from either population may plot within the overlapping area was then quantified using the respective cumulative distribution functions (CDFs). Discrimination efficiency was determined as: (probability of the non-overlapping area / 2) x 100%.

## Results and Discussion

### Single cell analysis

#### Oocyst selection and force measurement

**Fig 1:**
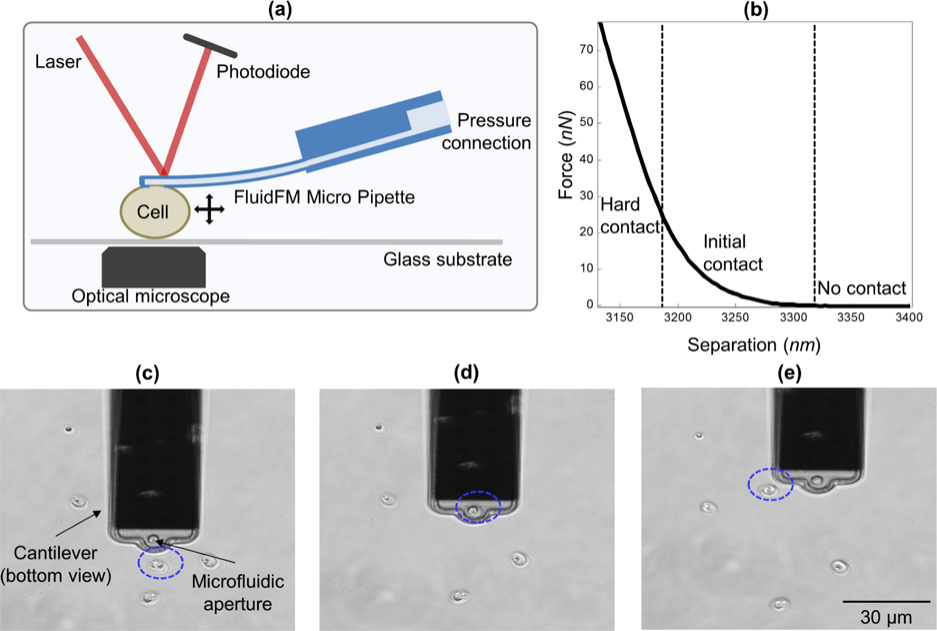
Oocyst measurement process **(a)** Schematic of FluidFM setup. **(b)** Typical force-distance curve showing zero (no contact), exponential (initial contact), and linear (hard contact) increase of force. **(c-e)** An oocyst (indicated by dashed blue oval) is selected, measured and released.

Oocysts were attracted and attached to the cantilever by applying suction pressure (typically 300 to 500 mbar). After a force measurement, the cantilever could be relocated to an arbitrary position and the oocyst was released by a pressure pulse up to 1000 mbar (Fig 1). In a single sample, over 50 oocysts were measured per hour when completing single measurements of each oocyst. Over 200 discrete measurements per hour were achieved when performing multiple measurements per oocyst. As *Cryptosporidium*-positive drinking-water samples typically contain between 1 and 90 oocysts per 1000 L filtered drinking-water [7], concentrated to 50 μL for detection, this in principle allows measuring the height and deformability of all subjects in a sample in approximately 1.5–2 hours, equivalent to the existing staining and microscopy process. We anticipate that a throughput of several samples per hour could be achieved by automating sample replacement and oocyst selection.

#### Force-distance curves

Illustrated in Fig 1B is an example force-distance curve comprising three stages: zero force when there is no contact, an exponential-like increase in force during initial contact and an apparent linear increase in force during hard contact between the oocyst and the glass surface. Tufenkji *et al*. [17] attributed the initial, exponential part of the force-distance curve to the glycoprotein layer which extends from the surface of the oocyst outer wall into the solution. We assume that the glycoprotein layer is fully collapsed during hard contact, and thereby attribute the final, linear part of the curve to the deformation of the oocyst body. Due to the high ionic strength of the buffer used (~ 0.15 mol L^-1^) the contribution of electrostatic repulsion between the oocyst and the glass surface is negligible (Debye screening length < 1 nm).

The deformation of the oocyst body can be approximated with a linear fit (average R^2^ > 0.99). The slope of this linear fit has the units of a spring constant (N/m). Though consistent with earlier reports [9,15], the physical basis for such a linear deformation is poorly understood: due to the oval shape of the oocysts, the contact area increases during compression, and one would rather expect a 3/2 power relationship, as in the Hertz model for the deformation of elastic spheres [25]. On the other hand, several assumptions in the Hertz model do not hold for biological objects. In particular, the oocyst is not a homogeneous substance; for instance, the fact that oocysts contain four sporozoites indicates a complex internal structure. In view of the lack of an underlying physical model, we use the obtained apparent spring constant value as an empirical parameter to quantify oocyst deformability.

Regarding the initial, exponential-like part of the force-distance curve, the glycoprotein layer of the oocyst might be modelled as a neutral polymer brush according to the de Gennes model [16,26]. Before applying such a model, the force-distance curves must be corrected for deformations of the oocyst body. However, during the initial contact phase a linear deformation of the oocyst body cannot be assumed, *e*.*g*., increase of contact area might play a significant role. In conclusion, during the initial contact phase the contributions of glycoprotein brush collapse and oocyst body deformation cannot be reliably separated. Therefore, we excluded this initial part of the force-distance curves from further analysis.

#### Multiple measurements per oocyst

**Fig 2:**
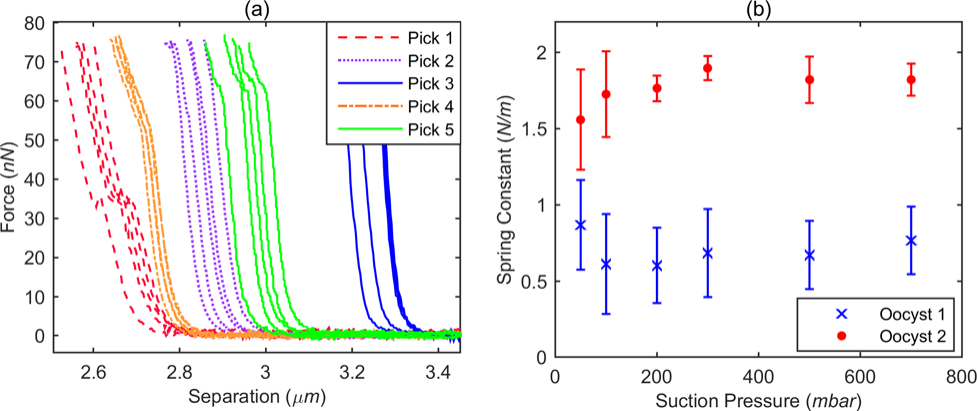
**(a)** Force-distance curves for five sets of five measurements on a single oocyst. A set of five measurements was taken whilst keeping the oocyst immobilised, and a single colour was assigned to the resulting force-distance curves; after release/re-immobilisation another set of five measurements was taken and a different colour assigned. **(b)** Effective spring constant values for two oocysts, obtained whilst attaching the oocyst to the cantilever using different suction pressures.

After measurement, an oocyst can be released undamaged (*i*.*e*., there is no systematic trend in subsequent measurements) from the cantilever aperture by a positive pressure pulse, and attracted again by a suction pressure to re-immobilise it on the cantilever. To assess the variability in spring constant and height for *C*. *parvum*, a series of experiments was performed where an oocyst was immobilised, measured five times and released, before the process was repeated another four times using the same subject. Due to the pressure-based release and attraction of the oocyst, the orientation in which it is attached to the cantilever is (quasi-)random each time it is immobilised. Displayed in Fig 2A is a series of force-distance curves obtained from a single untreated *C*. *parvum* oocyst, with each colour representing subsequent measurements without releasing the oocyst. The shape of the force-distance curves was highly reproducible when the oocyst was measured several times without releasing. This reproducibility indicates that oocyst deformation during force measurement is elastic rather than plastic. The curves within each set of measurements display random shifts of 50–100 nm in the apparent oocyst heights, which we attribute to mechanical drift. Whilst this amount of drift is relatively large for an AFM-based method, presumably due to the large mechanical loop between the sample and the cantilever in the FluidFM, it does not impact the analysis and conclusions in this study.

Fig 2A also shows that by immobilising and releasing the same subject several times, oocyst height estimations varied between ~2.7 μm and 3.3 μm due to orientation change. *Cryptosporidium* oocysts are not uniformly spherical in shape–oocysts are typically spheroidal, with one dimension larger than the other (*e*.*g*. *C*. *parvum* oocysts are reportedly 4.5 μm wide x 5.5 μm high) [10]. Therefore, if an oocyst is immobilised at the cantilever tip, its height measured, and then released, it is possible that a different height would be measured when repeating this process, should the same oocyst be immobilised in a different orientation. Measured height values are an underestimation of the physical dimensions of the oocyst, since oocysts were immobilised using a hollow aperture, which means that a portion of each oocyst was held within the cantilever. We conclude that our method can rapidly measure individual pathogens many times and in multiple orientations.

#### Kinks in force-distance curves

As seen in Fig 2A several force-distance curves displayed kinks, which were highly reproducible when multiple measurements were taken without releasing the oocyst. Kinks may be explained by a reversible change of the oocyst structure (*e*.*g*., buckling of the oocyst wall) under influence of the applied force, or, alternatively, by a reorientation of the oocyst at the aperture of the cantilever. Given the limited resolving power of optical microscopy, the type of structural transition that causes a kink cannot always be identified. Nevertheless, in some cases, we were able to correlate kinks with a rotational movement of the oocyst whilst applying force, as observed by optical microscopy during measurements (see S1 Video and S5 Fig). We found that curves with kinks provided a lower effective spring constant on average (S3 Fig).

#### Effect of suction pressure

During attraction and attachment of the oocyst, different suction pressures could be applied to the cantilever using the FluidFM pressure controller. To investigate whether the applied suction pressure impacted oocyst deformability, we subjected two separate oocysts to a number of suction pressures ranging from 50 to 700 mbar (Fig 2B). Each oocyst was measured up to eight times for each suction pressure. Between each measurement, the oocyst was released and reattached, in order to capture possible effects of oocyst orientation. The two oocysts showed different behaviour: one oocyst had high effective spring constant values with relatively small spread of the data, whilst for the other oocyst lower values and a larger spread were recorded. Additionally, this second oocyst appeared to be more prone to changes in orientation during measurement, as witnessed using optical microscopy and the presence of kinks in the force-distance curves. More generally, for both oocysts the measured spring constants did not depend on applied suction pressure, except possibly for the lowest suction pressures (50 and 100 mbar). Despite the absence of an effect of suction pressure on deformability, the frequency of kinks increased with decreasing suction pressure (along with a decrease in the average force threshold for these events to occur): it appears that it is easier to reorient an oocyst during measurement if it is held in place more loosely.

### Batch analysis

#### Inter-species variation in oocyst height and deformability

**Fig 3:**
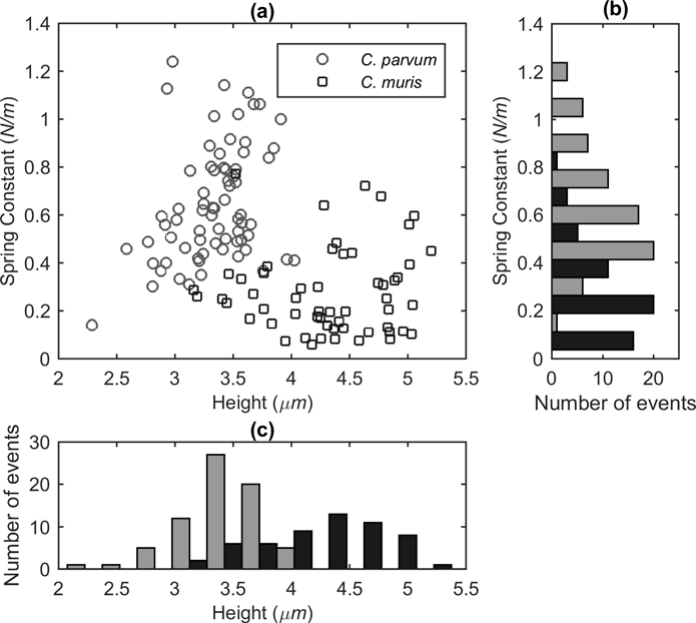
Height and deformability properties of untreated *C*. *muris* and *C*. *parvum* oocysts. **(a)** Height and spring constant data that were obtained for each single assayed oocyst of *C*. *muris* (*n* = 56) and *C*. *parvum* (*n* = 71) are plotted together in a scatterplot and are accompanied by side histograms, which show the distributions of the spring constant **(b)** and height **(c)** independently. The human pathogenic *C*. *parvum* is indicated by grey and *C*. *muris* is indicated by black.

Height and spring constant (*i*.*e*., the slope of the linear regime of the force-distance curves) values for untreated *C*. *muris* or *C*. *parvum* oocysts were extracted from the respective force spectroscopy measurements, which were obtained with a suction pressure of 300 mbar. A scatterplot (a) of oocyst height *vs*. spring constant values for each measured oocyst of *C*. *muris* and *C*. *parvum*, accompanied by histogram representations of spring constant (b) and height (c) distributions are displayed in Fig 3. These results imply that *C*. *muris* oocysts are generally more deformable (lower effective spring constant; median = 0.24 N/m) than *C*. *parvum* oocysts (median = 0.59 N/m). In addition, more variance existed in the height of *C*. *muris* than *C*. *parvum*, which is probably due to difference between the typical height and width of *C*. *muris* (on average 5.6 μm wide x 7.4 μm high) [10] being almost a factor of two larger than the same difference for *C*. *parvum* (4.5 μm x 5.5 μm) [10].

Using non-parametric probability density estimations for either the height or spring constant data of each species, it was possible to calculate a discrimination efficiency for either characteristic. Briefly, this was done by firstly fitting a kernel probability density function (PDF) to the height or spring constant data of each population, in order to define the overlapping region of the PDFs. Secondly, the probability that an oocyst is within this overlapping region was calculated using cumulative distribution functions (CDFs), to give an indication of the level of discrimination (*i*.*e*., the percentage of the total number of subjects that did not plot within the overlapping region). The discrimination efficiency, based on the measured height values, of *C*. *parvum* (median = 3.38 μm) and *C*. *muris* (median = 4.36 μm) was estimated at 86%, whereas 81% discrimination was calculated for spring constant data (See S2 Fig for overview of procedure). Although some overlap exists in the distributions of either height or spring constant data, the oocyst populations are almost completely distinct in the scatterplot of height *vs*. spring constant (Fig 3A), where discrimination is dependent on both characteristics.

Our findings suggest that deformability-based or size-based microfluidic separation systems may facilitate the separation of these species with an estimated 80–90% efficiency, but that near complete separation would be difficult to achieve. Instead, FluidFM could be used as a discrimination tool for *Cryptosporidium* species, which utilises both size and deformability properties to enable more efficient sorting than is possible with other methods. Sorting is possible because oocysts can be easily immobilised at the cantilever tip and thus can be removed from the sample or placed at a defined location. Given that the “major” human-pathogenic species (*C*. *parvum*, *C*. *hominis*, *C*. *meleagridis*, *C*. *canis*, *C*. *cuniculus*, *C*. *felis*) are very similar in size (~4.0–5.5 μm) [27], analysis could potentially be performed to discriminate between oocysts of the “major” species and others that are out with this size range (*e*.*g*., *C*. *xiaoi*, *C*. *muris*, *C*. *andersoni*, *C*. *baileyi*).

#### Effects of temperature-based inactivation treatment on *C*. *parvum* height and deformability

**Fig 4:**
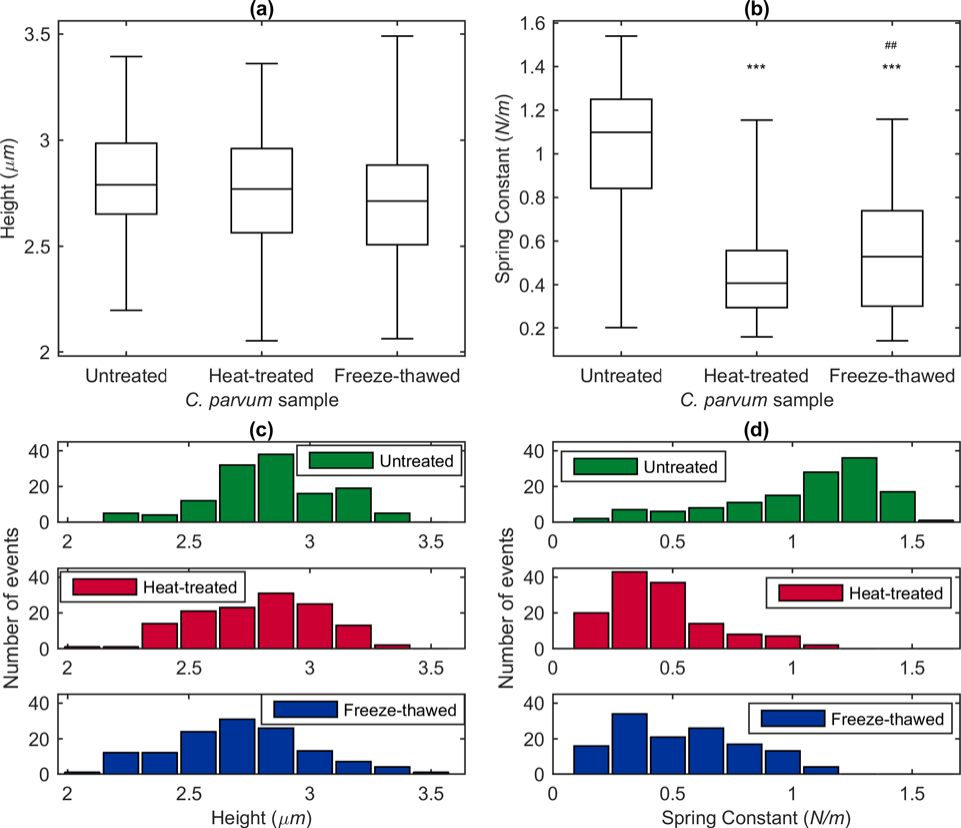
Height and spring constant data for untreated, heat-treated and freeze-thawed *C*. *parvum* oocysts obtained using FluidFM. Height **(a)** and spring constant **(b)** data for untreated (*n* = 184), heat-treated (*n* = 224) and freeze-thawed *C*. *parvum* oocysts (*n* = 131) are represented by boxplots which display medians and quartiles for the respective distributions; the upper and lower error bar caps are indicative of the maximum and minimum recorded values. *** Significantly different from untreated group (*p* < 0.001). ^##^ Significantly different from heat-treated group (*p* < 0.01). The distributions of height **(c)** and spring constant **(d)** are presented using histograms.

Height and spring constant distributions for untreated, heat-treated and freeze-thawed sub-populations of *C*. *parvum* are presented in Fig 4. For each sub-population, 30 individual oocysts were measured in up to eight orientations using a suction pressure of 500 mbar. The oocyst height distributions of untreated, heat-treated and freeze-thawed *C*. *parvum* are presented in Fig 4A. Considerable overlap exists between the height distributions of heat-treated, freeze-thawed and untreated *C*. *parvum* (Fig 4C), demonstrating that size does not provide a marker for viability.

Spring constant distributions for each sub-population are presented in Fig 4B. Generally, these results indicate that untreated oocysts are less deformable than heat-treated and freeze-thawed oocysts. Increased deformability in temperature-inactivated oocysts might be caused by breakdown of internal structures, for example membranes and organelles of the four sporozoites contained within an oocyst. Alternatively, the structure of the oocyst outer wall may have been altered by the inactivation treatment. The *C*. *parvum* oocyst outer wall is a trilaminar structure and the inner [28] and central [29] layers are considered to contribute to the elasticity exhibited by the oocyst wall. Upon temperature-induced loss of viability, the proteins responsible for elasticity within these layers may have become degraded and/or denatured.

A statistically significant difference between the deformability distributions of the oocyst sub-populations was indicated by the Welch’s F test; *F* (2,290) = 192.62, *p* < 0.001. Furthermore, *post-hoc* means analysis using the Games-Howell test revealed a statistically significant reduction in spring constant for heat-treated (mean difference = 0.56 N/m, *p* < 0.001) and freeze-thawed (mean difference = 0.46 N/m, *p* < 0.001) oocysts when compared to untreated oocysts (mean = 1.03 N/m). We calculated a discrimination efficiency of 85% for the spring constant distributions of untreated and heat-treated oocysts. In addition, untreated and freeze-thawed oocysts were discriminated with an efficiency of 78% based on spring constant data. Despite being comparably lower, a statistically significant difference was also measured between the spring constants of heat-treated and freeze-thawed oocysts using a Games-Howell test (mean difference = 0.10, *p* < 0.01), indicating that the method of inactivation also somewhat influences deformability. A discrimination efficiency of less than 20% was calculated for the spring constant distribution of heat-treated and freeze-thawed *C*. *parvum*.

Presented in Fig 4D are histogram representations of the spring constant distributions for untreated and temperature-inactivated *C*. *parvum*. The histogram of untreated *C*. *parvum* data has two peaks–the smaller, low spring constant (high deformability) peak corresponds to the peak of the temperature-inactivated populations, providing evidence for the existence of a smaller, naturally occurring, non-viable subpopulation within the untreated sample. These data indicate an 86% viability rate (approximated using the corresponding PDF; see S4 Fig), which agrees with the 92% viability rate calculated by the supplier for this sample upon completion of a conventional excystation assay [30].

## Conclusions and Outlook

We present a novel force spectroscopy method which allows single-oocyst immobilisation, manoeuvring, and force measurement of isolated subjects at a rate of > 50 subjects per hour, but with capacity for > 200 discrete measurements per hour. Our results reveal height and deformability distributions for untreated and temperature-inactivated *C*. *parvum*–after inactivation *C*. *parvum* appears to have increased deformability. Though differences in deformability between these populations were statistically significant, they were also overlapping (as were the respective height distributions), implying that deformability-based (and size-based) separation methods, *e*.*g*., microfluidic systems, cannot be used for the complete sorting of viable and non-viable sub-populations of *C*. *parvum*. However, for species determination, the use of force spectroscopy allows measurement of height and deformability synchronously, and when effective height and spring constant data were plotted together, this permitted the efficient discrimination of *C*. *parvum* and *C*. *muris*.

Future work should address inter-batch variability, ageing and environmental influences (*e*.*g*., UV light). To confirm our finding that increased deformability correlates with inactivation, the FluidFM can be used to sort oocysts in sub-populations with high or low deformability, followed by animal infectivity assays. Finally, the methods presented in this paper may be applied to other waterborne pathogens and single-celled organisms to reveal their biomechanical properties.

## Supporting Information

S1 Fig Data Analysis Procedure. (PDF)

S2 Fig Discrimination Efficiency Estimation. (PDF)

S3 Fig Presence of Kinks in Force-Distance Curves. (PDF)

S4 Fig Probability Density Functions Fitted to *Cryptosporidium parvum* Spring Constant Data. (PDF)

S5 Fig Force-Distance Curve Corresponding to S1 Video. (PDF)

S1 Video Rotational Movement of Oocyst During Measurement. (MP4)
