# Supplementary material for: Deformability Assessment of Waterborne Protozoa Using a Microfluidic-Enabled Force Microscopy Probe
Source: PLoS One. 2016 Mar 3;11(3):e0150438. doi: 10.1371/journal.pone.0150438 (PMC4777494; doi:10.1371/journal.pone.0150438)
Supplement: S1 Fig — (PDF) [file pone.0150438.s001.pdf]

S1 Figure: Data Analysis Procedure

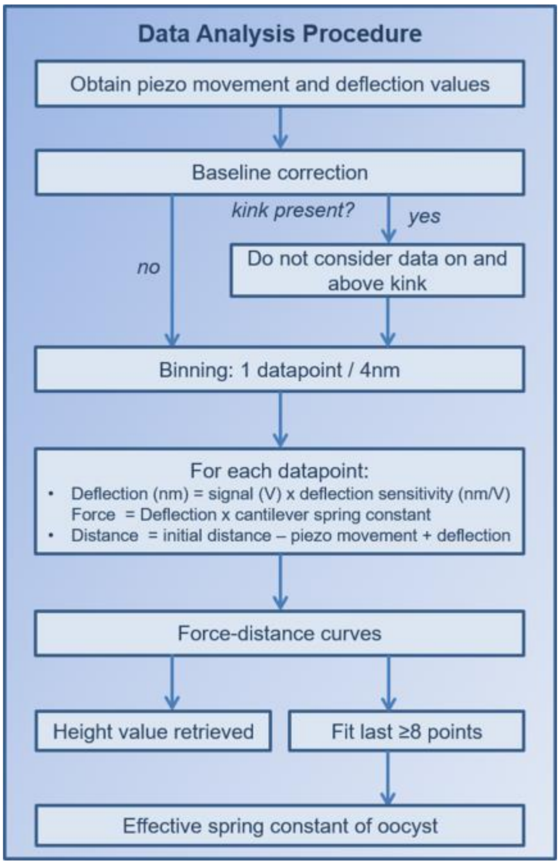

S1: Post-measurement data processing for estimating the biomechanical properties of a single oocyst. We corrected for linear drift of the baseline during approach. For linear fitting, we iterated over the last *n* points ($n \geq 8$) of the force-distance curves, calculating the R^2-value (RSQ) of the fit for each *n*. Then, for each *n* a fit quality parameter $fq(n) = (\mathrm{RSQ}(n) - 0.995) * n$ was calculated and we choose the fit with the largest value of $fq(n)$. We considered this value a reasonable trade-off between a high R^2-value for the linear fit and a maximum number of points to be included in the final (near)-linear part of the force-distance curves.
