# Supplementary material for: Deformability Assessment of Waterborne Protozoa Using a Microfluidic-Enabled Force Microscopy Probe
Source: PLoS One. 2016 Mar 3;11(3):e0150438. doi: 10.1371/journal.pone.0150438 (PMC4777494; doi:10.1371/journal.pone.0150438)
Supplement: S2 Fig — (PDF) [file pone.0150438.s002.pdf]

S2 Figure: Discrimination Efficiency Estimation

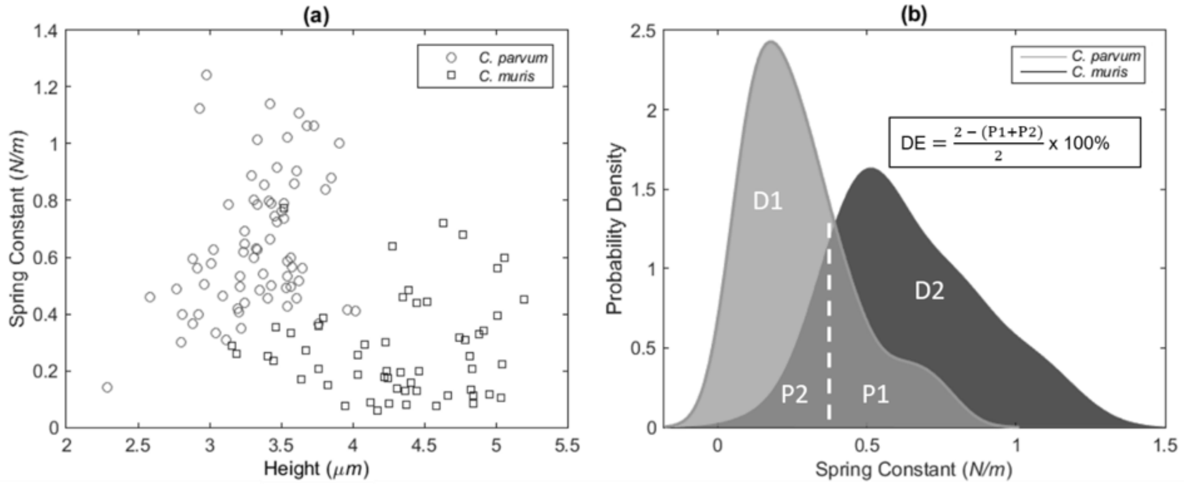

S2: Calculation of discrimination efficiency between two populations. **(a)** Effective spring constant and height values were calculated for populations of *C. parvum* (light grey circles) and *C. muris* (black squares). **(b)** Using spring constant data as an example: the discrimination efficiency (DE) was estimated by firstly calculating the probability density function (PDF) of *C. parvum* (light grey) and *C. muris* (black) data; non-parametric PDFs (*i.e.*, Kernel PDFs) were used due to skewness (non-normality) of data distribution. The probability of finding a *C. muris* oocyst within the *C. parvum* population (P1) plus the probability of finding a *C. parvum* oocyst within the *C. muris* population (P2) was then calculated; this is the probability of the overlapping area. Finally, the DE was calculated by finding the percentage of the total area which was completely discrete (D1 and D2); DE = (2 - (P1 + P2)) / 2 x 100%.
