# Supplementary material for: Deformability Assessment of Waterborne Protozoa Using a Microfluidic-Enabled Force Microscopy Probe
Source: PLoS One. 2016 Mar 3;11(3):e0150438. doi: 10.1371/journal.pone.0150438 (PMC4777494; doi:10.1371/journal.pone.0150438)
Supplement: S3 Fig — (PDF) [file pone.0150438.s003.pdf]

S3 Figure: Presence of Kinks in Force-Distance Curves

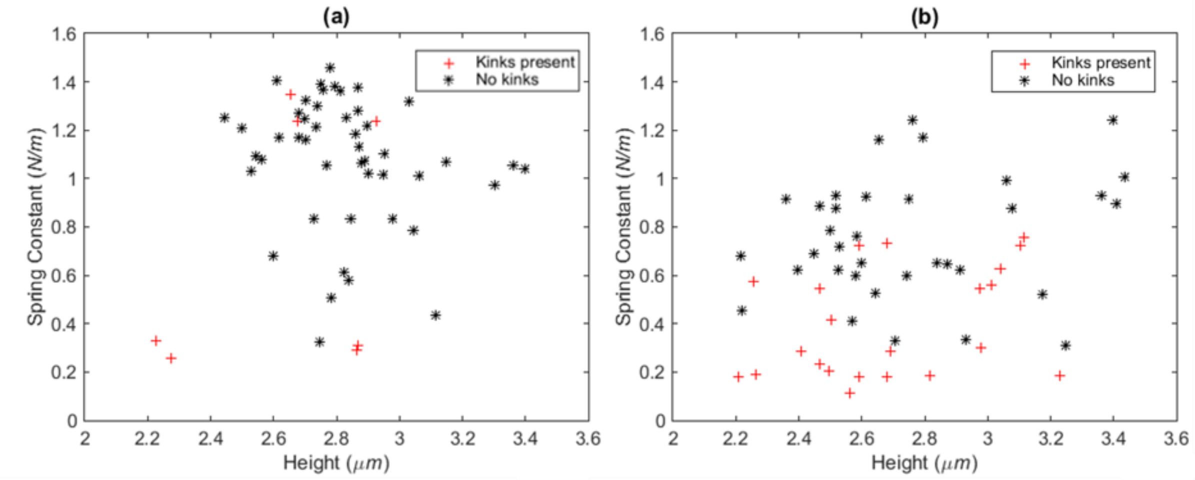

S3: Occurrence of kinks in force-distance curves. Distribution of effective spring constant *vs*. height values for untreated **(a)** and freeze-thawed **(b)** *C. parvum*; the relative occurrence of kinks (indicated by red '+') is higher for lower apparent spring constants, possibly because of a sliding movement of the oocysts during the measurement.
