# Supplementary material for: Deformability Assessment of Waterborne Protozoa Using a Microfluidic-Enabled Force Microscopy Probe
Source: PLoS One. 2016 Mar 3;11(3):e0150438. doi: 10.1371/journal.pone.0150438 (PMC4777494; doi:10.1371/journal.pone.0150438)
Supplement: S4 Fig — (PDF) [file pone.0150438.s004.pdf]

S4 Figure: Probability Density Functions Fitted to *Cryptosporidium parvum* Spring Constant Data.

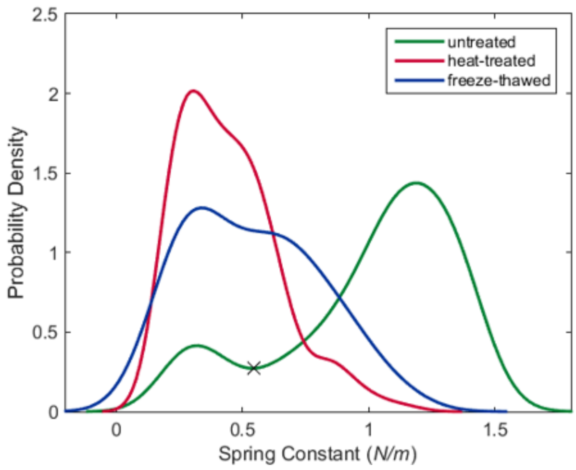

S4: Probability density estimation of spring constant data for untreated and temperature-inactivated *C. parvum*. Spring constant data has been fitted with a kernel probability density function (PDF) – a non-parametric estimation of the PDF – due to data skewness. For untreated *C. parvum*, the smaller peak of the PDF occurs at similar spring constant values to the peaks of the PDFs for temperature-inactivated oocysts. Sample viability of the untreated sample was estimated at 86% due to 14% of oocysts being distributed to the left side of the minima (indicated by black cross) between the two peaks of the PDF.
