# Supplementary material for: Deformability Assessment of Waterborne Protozoa Using a Microfluidic-Enabled Force Microscopy Probe
Source: PLoS One. 2016 Mar 3;11(3):e0150438. doi: 10.1371/journal.pone.0150438 (PMC4777494; doi:10.1371/journal.pone.0150438)
Supplement: S5 Fig — (PDF) [file pone.0150438.s005.pdf]

S5 Figure: Force-Distance Curve Corresponding to SI Video.

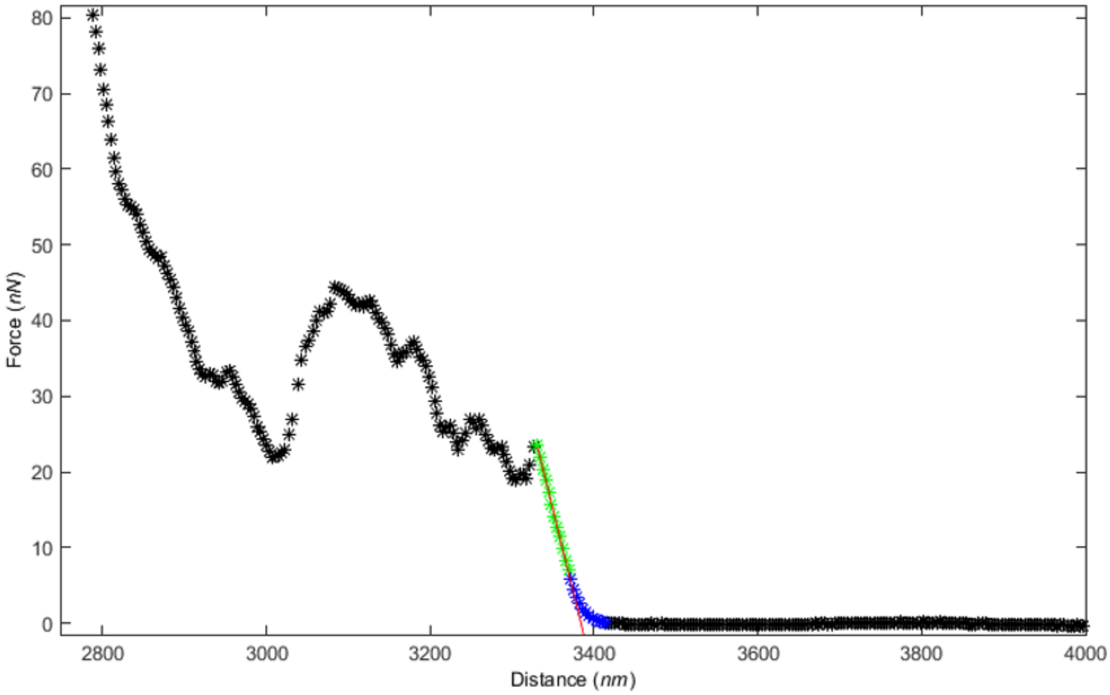

S5: Force-distance curve corresponding to SI video. A large kink is present, correlating to the rotational movement observed in the video. The red line is a linear fit representing the effective spring constant of the oocyst during hard contact. The data on and above the kink were excluded from the fitting procedure. Green points are fitted points and correspond to hard contact. Blue points correspond to the early, exponential-like contact phase. The rightmost blue point (force starts to exceed a 0.1 nN threshold) indicates the measured oocyst height.
